# Towards Autonomous Robotic Biopsy—Design, Modeling and Control of a Robot for Needle Insertion of a Commercial Full Core Biopsy Instrument

**DOI:** 10.3389/frobt.2022.896267

**Published:** 2022-06-15

**Authors:** Seyed MohammadReza Sajadi, Seyed Mojtaba Karbasi, Henrik Brun, Jim Tørresen, Ole Jacob Elle, Kim Mathiassen

**Affiliations:** ^1^ The Research Group of Robotics and Intelligent Systems (ROBIN), Department of Informatics, University of Oslo, Oslo, Norway; ^2^ Digital Signal Processing Group, Department of Informatics, University of Oslo, Oslo, Norway; ^3^ RITMO Centre for Interdisciplinary Studies in Rhythm, Time and Motion, University of Oslo, Oslo, Norway; ^4^ The Intervention Centre, Oslo University Hospital, Oslo, Norway; ^5^ The Department for Pediatric Cardiology, Oslo University Hospital, Oslo, Norway; ^6^ Department of Technology Systems, University of Oslo, Oslo, Norway

**Keywords:** percutaneou needle biopsy, robot-assisted, medical robotic, reinforcement learning, deep deterministic policy gradient, ultrasound guided robotic biopsy, mechanical design, autonomous system

## Abstract

This paper presents the design, control, and experimental evaluation of a novel fully automated robotic-assisted system for the positioning and insertion of a commercial full core biopsy instrument under guidance by ultrasound imaging. The robotic system consisted of a novel 4 Degree of freedom (DOF) add-on robot for the positioning and insertion of the biopsy instrument that is attached to a UR5-based teleoperation system with 6 DOF. The robotic system incorporates the advantages of both freehand and probe-guided biopsy techniques. The proposed robotic system can be used as a slave robot in a teleoperation configuration or as an autonomous or semi-autonomous robot in the future. While the UR5 manipulator was controlled using a teleoperation scheme with force controller, a reinforcement learning based controller using the Deep Deterministic Policy Gradient (DDPG) algorithm was developed for the add-on robotic system. The dexterous workspace analysis of the add-on robotic system demonstrated that the system has a suitable workspace within the US image. Two sets of comprehensive experiments including four experiments were performed to evaluate the robotic system’s performance in terms of the biopsy instrument positioning, and the insertion of the needle inside the ultrasound plane. The experimental results showed the ability of the robotic system for in-plane needle insertion. The overall mean error of all four experiments in the tracking of the needle angle was 0.446°, and the resolution of the needle insertion was 0.002 mm.

## 1 Introduction

Percutaneous liver biopsy (PLB) is the gold standard procedure in the diagnosis of parenchymal liver disease and focal hepatic lesions (Al Knawy and Shiffman, 2007). While various non-invasive methods have been developed and are now accessible, liver biopsy continues to play an essential role in the diagnosis of liver disease (Lim and Kim, 2020). Despite the fact that PLB has evolved as a result of scientific advances in imaging technology and biopsy equipment, there are still some shortcomings with manual biopsies that could be addressed by a more stabilized and dexterous robotic system compared to human hands (Siepel et al., 2021). In addition, a robotic system for PLB that can be employed in a teleoperation configuration could be useful to address the lack of expert radiologists in remote areas.

PLB involves inserting a thin needle through the abdomen into the liver and removing a tissue sample from a suspected lesion for further pathological examination. Freehand biopsy and probe-guided biopsy are the two most commonly used techniques in manual biopsy procedures. While flexibility to choose the best needle path is the primary advantage of the freehand biopsy, maintain the needle within the 2D ultrasound image is challenging. The probe-guided biopsy limits the radiologist’s flexibility to choose the needle path by attaching the needle to the ultrasound probe to keep the needle within the ultrasound image (Phal et al., 2005). A discrepancy between the biopsy specimen and the target tissue due to incorrect biopsy needle insertion can lead to misinterpretation and errors in the diagnosis (Su et al., 2021). Therefore, the procedure’s quality is determined by the radiologist’s expertise, precision, and dexterity.

The size and quality of the specimen are the other crucial factors in a successful biopsy. The quality and physical features are critical for the diagnostic informative value of a PLB to reduce the risk of misinterpretation and enhance the inter-observer variability. In general, core biopsy needles are classified into two types: side-notch needles and end-cutting needles. The quality and physical features of the specimen of an end-cut full core biopsy instrument have been compared with a side-notch biopsy instrument for 32 liver biopsies in (Schaible et al., 2020). The study confirmed the superiority of full-core biopsy instrument over side-notch needles in terms of specimen diameter, fragmentation, and overall diagnostic value.

We believe that by developing a robotic biopsy system based on an end-cut full core biopsy instrument, the informative diagnostic quality of the specimen could be improved, and the robotic system can incorporate the advantages of both freehand and probe-guided biopsy techniques. Such a robotic system is well suited for use as a slave robot in a teleoperation configuration, where decreasing the risk of misinterpretation and increasing inter-observer variability are critical. Furthermore, a combination of machine learning and robotics can offer superior dexterity, pointing the way toward autonomous or semi-autonomous robotic biopsy in the future by offering more precise biopsies and tumor detection.

Several robotic systems for robotic-assisted biopsy developed for various anatomical locations and imaging modalities. Robotic systems for PLB have been developed using different imaging modalities, such as computed tomography (CT) (Cornelis et al., 2015; Won et al., 2017; Ben-David et al., 2018; Heerink et al., 2019), magnetic resonance imaging (MRI) (Stoianovici et al., 2013; Song et al., 2013; Hiraki et al., 2018) and, ultrasound imaging (US) (Mignon et al., 2018). The robotic systems described in (Song et al., 2013; Franco et al., 2015; Heerink et al., 2019), were developed to place the needle, while the systems described in (Won et al., 2017; Ben-David et al., 2018) were designed for needle positioning and insertion. Few robotic systems have been developed based on full core biopsy instruments. A three degree of freedom MRI-safe robot for prostate biopsy based on a biopsy gun has been reported in (Stoianovici et al., 2013). The robotic system designed for the needle positioning while the needle insertion and firing the gun are still manual.

A comprehensive overview of recent robotic-assisted percutaneous solutions, as well as an explanation of the key workflow phases and design elements of robotic-assisted percutaneous solutions presented in (Siepel et al., 2021). The workflow and design elements are considered in the development of an end-effector (EE) for use in a robot-assisted breast biopsy (Welleweerd et al., 2020). The end-effector includes a 3 DOF needle guide, and a mechanism for stopping the needle, which is mounted on a 7 DOF robotic manipulator. In this design the needle is controlled in all degrees of freedom except for the direction of insertion, which is manual and controlled by the radiologist.

To the best of our knowledge, there is a knowledge gap in developing a fully automated ultrasound-guided robotic system for liver biopsy based on full-core biopsy instruments. This paper aims to design and develop a fully automated robotic-assisted system for the positioning and insertion of a commercial end cut full core biopsy instrument. The proposed robotic system can be used as a slave robot in a teleoperation configuration or as an autonomous or semi-autonomous robot in the future. The system’s design objectives are manifold: eliminate tremor of radiologist’s hand by developing a more stabilized robotic system with higher stiffness and precision, enhancing the informative diagnostic quality of the specimen by using a commercial core needle biopsy instrument, incorporating the advantages of both freehand and probe-guided biopsy techniques, reduce procedure time, reduce radiologist hand-eye coordination requirements, work as a slave robot in a teleoperation configuration, and be ready for use as an autonomous or semi-autonomous system.

The proposed robotic system in this paper consists of a novel 4 DOF add-on robotic system and a UR5-based teleoperation system with 6 DOF. The UR5 manipulator is controlled by a radiologist utilizing a master-slave configuration and force controller to manipulate the ultrasound probe and the add-on robotic system that was developed previously by the authors in (Mathiassen et al., 2016). The add-on robotic system is controlled by a reinforcement learning-based controller (RL) using the Deep Deterministic Policy Gradient (DDPG) algorithm to position the instrument in the ultrasound plane based on inputs from the radiologist.

Our motivation to develop the RL-based controller instead of a classic controller is to prepare the robotic system for further upgrades by integrating the ultrasound images as sensory information for an envisioned autonomous robotic biopsy based on the ultrasound image in the future. Therefore, we develop the RL-based controller based on inputs from the radiologist as the first step to achieving this objective in this paper. In this way, the performance of the RL-Agent in a simpler environment in comparison to image-based reinforcement learning can be investigated.

The remainder of this paper is structured as follows: Section 2 presents the conceptual design based on clinical requirements. The engineering design and prototyping of the proposed robotic system are described in Section 3. The proposed RL-based controller is presented in Section 4. Finally, the proposed robotic system and controller are validated through four comprehensive experiments in Section 5.

## 2 Conceptual Design Based on the Clinical Requirements

The design approach to develop the robotic system in this paper is based on a commercial full-core-biopsy instrument. The key advantages of this approach are decreasing the design time and prototyping costs, enhancing fault tolerance, and system’s adaptability to be employed either as a teleoperation system or a semi-autonomous system (Brunete et al., 2017). Conceptual design based on clinical requirements is the initial phase of the design process. The objective is to develop the robotic system as close to the clinical scenario as feasible while the system incorporates the advantages of robotics, such as enhanced dexterity and precision (Sajadi et al., 2022).

The superiority of full-core-biopsy instruments (FC) over side-notch needles (SN) has been verified in term of the diameter of the specimen, fragmentation, and overall diagnostic value. The quality and physical characteristics of the specimen from a FC biopsy needle and a standard SN needle for liver biopsies of 32 individuals were compared in (Schaible et al., 2020). The study found that the FC-group had considerably higher specimen quality, with an average value of 1.68 vs. 2.50 (*p* = 0.009). Additionally, the fragmentation rate was statistically substantially lower in the FC-group at 2/27 (7%) than in the SN-group at 13/33 (39%) (*p* = 0.021) (Schaible et al., 2020).

**FIGURE 1:**
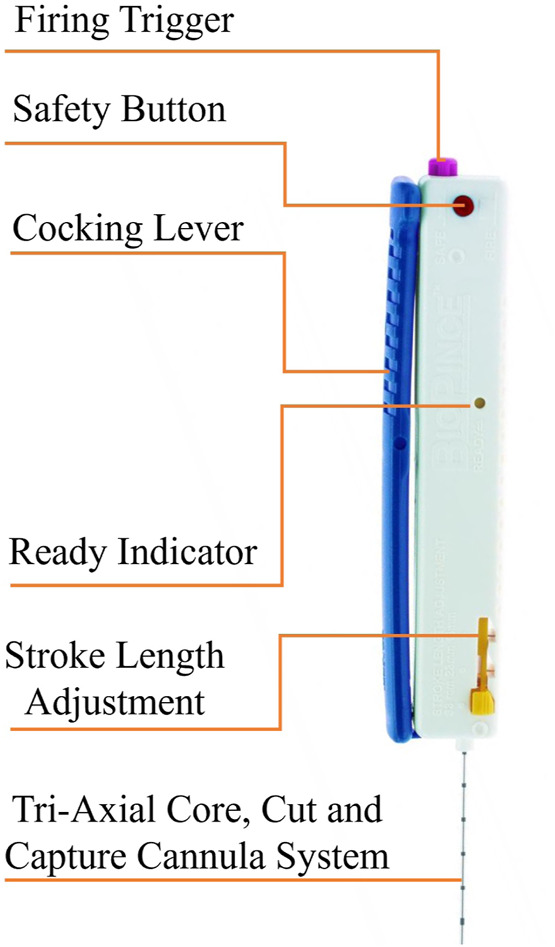
The main components of the Full Core Biopsy Instrument.

Figure 1 illustrates main components of a BioPince full core biopsy instrument which is used in this paper. The needle of this device is 200 mm in length and has three different throw lengths to increase clinical flexibility where 13 mm throw results in a specimen length of 9 mm, 23 mm throw results in a specimen length of 19 mm, and 33 mm throw results in a specimen length of 29 mm. During manual ultrasound-guided percutaneous liver biopsies, the clinician uses ultrasound images to determine the optimal location for inserting the needle. After that, the biopsy needle is guided by ultrasound, either by freehand biopsy or probe-guided biopsy techniques during the procedure (Rockey et al., 2009). Curved array probes are the most commonly used ultrasound probes during percutaneous liver biopsies.

The conceptual design aims to develop the robotic system in such a way that the system takes the advantage of both freehand and probe-guided biopsies techniques in term of a fully automated needle insertion system. The design should also cover all required DOFs to be employed as a slave robot in a long-distance teleoperation system or as an autonomous or semi-autonomous robot in the future. The system’s design objectives are manifold: 1) Fully automate a commercial full-core biopsy instrument, 2) Robotic needle insertion in the US plane without reducing the flexibility of the insertion path to a fixed position, respect to the ultrasound probe, 3) Eliminate tremor of radiologist’s hand, 4) Improve the accuracy and reduce the procedure time, 5) Reduce radiologist hand-eye coordination requirements, 6) work as a slave robot in a teleoperation configuration, and be ready for use as an autonomous or semi-autonomous system.

During an envisioned robotic percutaneous image-guided procedure, the clinician first moves the ultrasound probe that is directly attached to the teleoperated UR5 manipulator, to detect the target tissue, and then the add-on robotic system inserts the needle in the ultrasound image plane (in plane) to collect a sample of the tissue in the US image.

**FIGURE 2:**
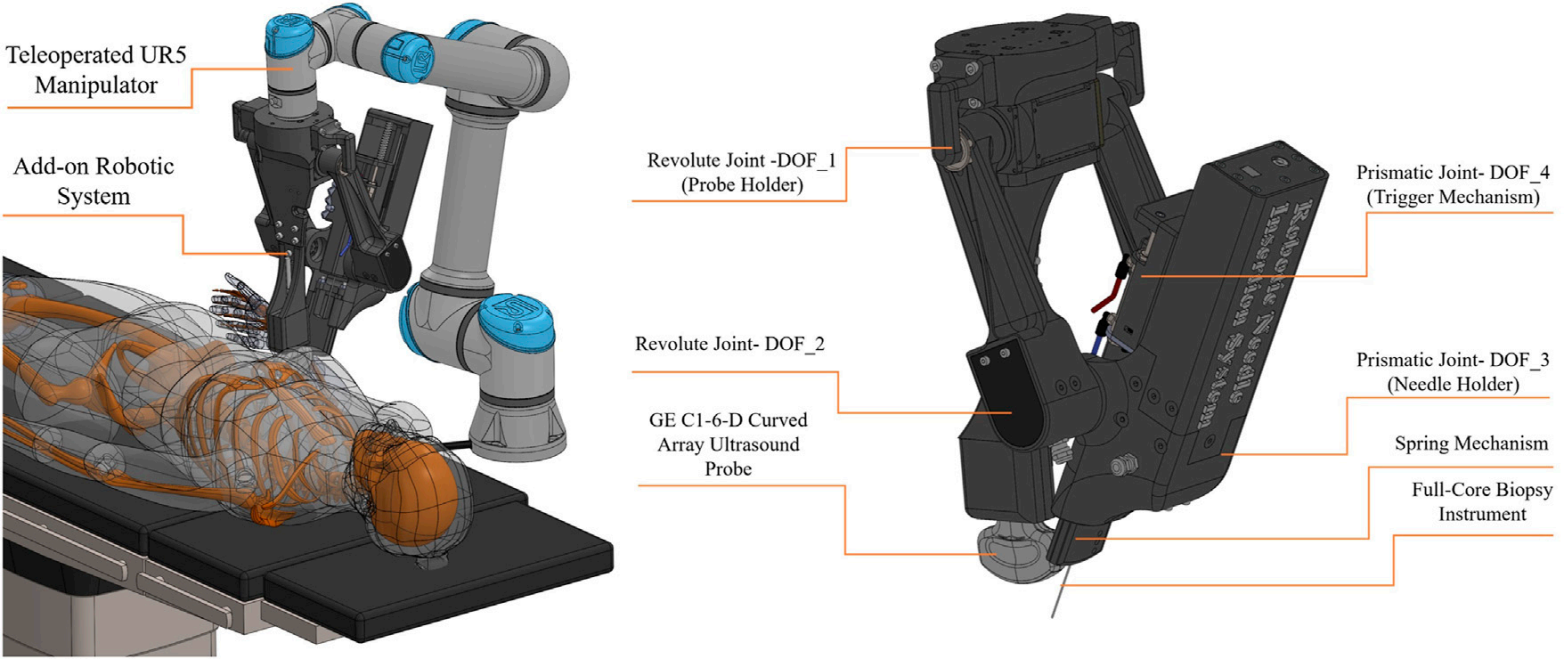
Conceptual design- hardware architecture.

Figure 2 illustrates the hardware architecture of the proposed robotic system. The proposed robotic system consists of an add-on robotic system and a UR5-based teleoperation system. The add-on robotic system is a novel 4 DOF customized robot for a commercial core needle biopsy instrument that is integrated with an ultrasound probe as depicts in Figure 2. The needle is positioned in a desired position inside the US plane using two revolute and one prismatic joint actuated by high resolution servomotors. There is an additional DOF for triggering the needle gun to take a sample from the target tissue. A spring mechanism in the add-on robotic system guides the needle and presses the skin’s surface before the insertion to prevent high deflection in the needle. The add-on robot is attached to the end-effector of the UR5 manipulator.

**FIGURE 3:**
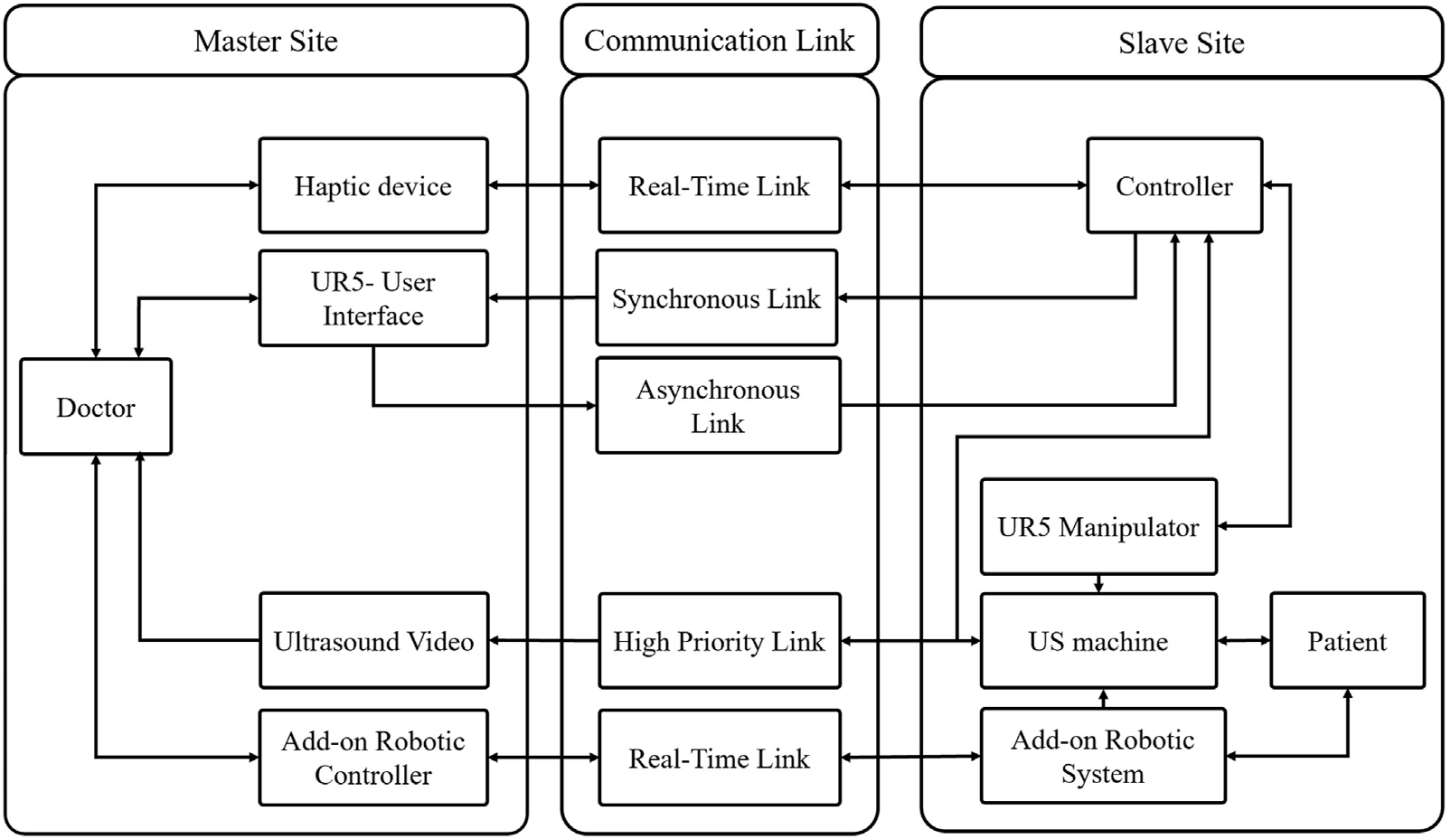
Conceptual design- software architecture.

Figure 3 illustrates the software architecture of the proposed robotic system. The UR5 manipulator is controlled by a radiologist utilizing a master-slave configuration and a human-in-the-loop control strategy. A 6 DOF force/torque sensor is included in the teleoperation system, and a force controller is used to maintain a constant pressure between the US probe and the patient’s body. The add-on robotic system is controlled by a RL-based controller using the Deep Deterministic Policy Gradient (DDPG) algorithm that is suitable for continuous action spaces. Since the authors have previously discussed the stability of the teleoperation system and the force controller in (Mathiassen et al., 2016), this work focuses on the mechanical design, workspace analysis, and control of the add-on robotic system.

## 3 Engineering Design and Prototyping

**FIGURE 4:**
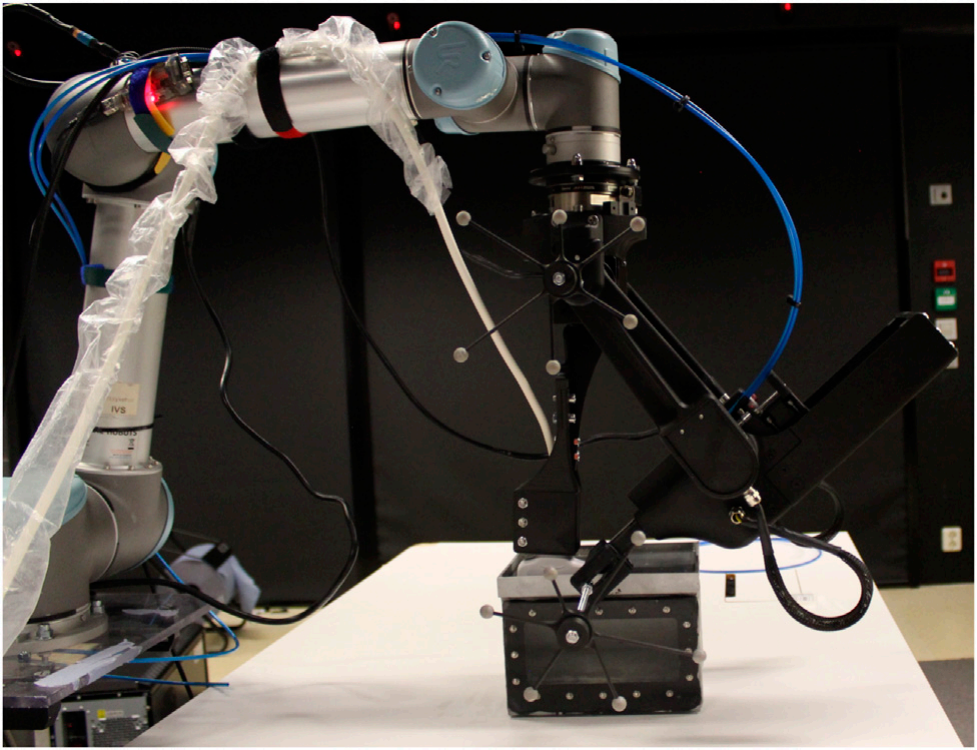
The prototype of the proposed robotic system in this paper.

The primary objectives of the engineering design are simplicity and rapid prototyping. Rapid prototyping is achieved by a combination of smart actuators, mechanical modules, and additive manufacturing. Figure 4 is illustrated the prototype of the proposed robotic system in this paper. The engineering design, as well as the mechanism for each degree of freedom of the add-on robotic system, is explained as follows:

### 3.1 Revolute Joint-1 (Probe Holder)

**FIGURE 5:**
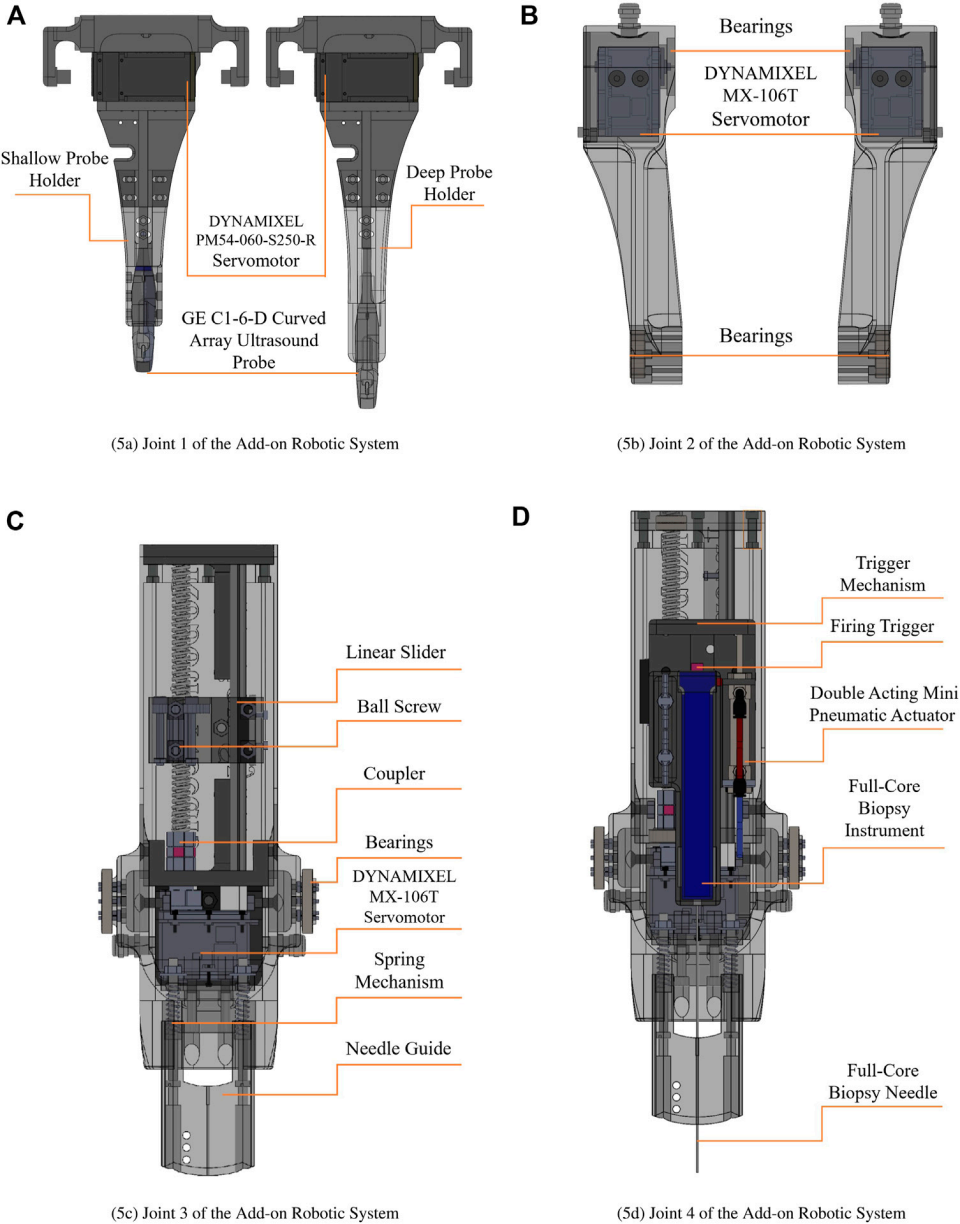
Engineering design of the add-on robotic system. **(A)** Joint 1 of the Add-on Robotic System, **(B)** Joint 2 of the Add-on Robotic System, **(C)** Joint 3 of the Add-on Robotic System¸ **(D)** Joint 4 of the Add-on Robotic System.

This degree of freedom is the base joint of the add-on robotic system where the ultrasound probe is attached. A GE C1-6-D curved array ultrasound probe is used in the development of the robotic system in this paper. This degree of freedom is directly connected to the UR5 manipulator’s end-effector, whereas the joint manipulates the first link. The first link consists of two parallel links; one is active and is directly actuated by a DYNAMIXEL PM54-060-S250-R servomotor, while the other is passive and is supported by a set of bearings. There is a 5 mms gap between the ultrasonic probe holder and the base joint for calibration reasons, in order to align the 2D ultrasound image plane with the needle cross section plane. This degree of freedom is illustrated at Figure 5A.

### 3.2 Revolute Joint-2

Two MX-106 DYNAMIXEL servo motors are directly attached at the end of passive and active links. These motors operate in dual mode which is combining two DYNAMIXEL servo motors into a single joint to increase the joint’s performance via increased output torque. In the dual mode configuration, one motor serves as the master and the other as the slave, and they are linked through a three-wired synchronization connection. It means that we send a single control signal to the master servomotor and the slave servo motor follows the control signal in opposite direction. The revolute joint-2 actuates the main body of the add-on robotic system which houses the needle holder, prismatic joint, pneumatic trigger, and spring mechanism. This degree of freedom is illustrated at Figure 5B.

### 3.3 Prismatic Joint-3 (Needle Holder)

Figure 5C shows the mechanism for this degree of from, that consist of a DYNAMIXEL MX-64T servomotor with a ball screw mechanism which is supported by a linear slider. The ball screw mechanism has a 200 mm stroke and a 10 mm lead. Considering the resolution of the servomotor, which is 4,096 pulses/revolution, the resolution of the prismatic joint is 0.002 mm/pulse. It implies that the robotic system inserts and stops the needle with incredible precision. The needle holder and pneumatic trigger are mounted at the top of this degree of freedom. There is a 10 mms gap in the needle holder for calibration reasons to alien the 2D ultrasound image plane with the needle cross section plane.

### 3.4 Prismatic Joint-4 (Pneumatic Trigger)

The full core biopsy instrument is equipped with a firing trigger that activates the cut and capture cannula system for taking the sample from the target tissue. A double acting mini pneumatic cylinder with a 30 mm stroke is employed as the actuator for this degree of freedom. Figure 5D illustrate this degree of freedom.

### 3.5 Work-Space Analysis of the Add-On Robotic System

The dexterous workspace of the robotic system is investigated to identify an unobstructed zone in which the physician may perform the biopsy operation. The dexterous workspace is a collection of point in the workspace that can be accessible from any arbitrary orientation (Sciavicco et al., 2011), or more precisely, a subset of point that can be used by a physician. Considering the free-hand technique, the desired workspace for the robotic system includes the region that the physician can manipulate the ultrasound probe with any arbitrary orientation and the area inside the ultrasound image that the needle can reach. The authors previously conducted a workspace analysis for the teleoperated UR5 manipulator in (Mathiassen et al., 2016). This section is devoted to analyzing the dexterous workspace inside the ultrasound image for the add-on robotic system.

To improve the flexibility of needle insertion, the add-on robot requires an additional degree of freedom. However, since the robot is designed around a full core biopsy instrument rather than simply a needle, adding another DOF increases the weight of the add-on robotic system. If the weight of the add-on robot exceeds 5kg, the UR5 manipulator will no longer be able to hold and manipulate it, given the UR5’s maximum payload of 5 kg. On the other hand, if we used a manipulator with a higher payload, the manipulator would no longer be lightweight, and the system’s footprint would be large, making it unsuitable for an operating room.

To address above challenge a novel passive spring mechanism has been designed to enhance the workspace of the system within the US image. The spring mechanism allows the length of the link-2 to be adjustable between 160 and 200 mm. Furthermore, the spring mechanism in the add-on robotic system guides the needle and presses the skin’s surface before the insertion to prevent high deflection in the needle.

**FIGURE 6:**
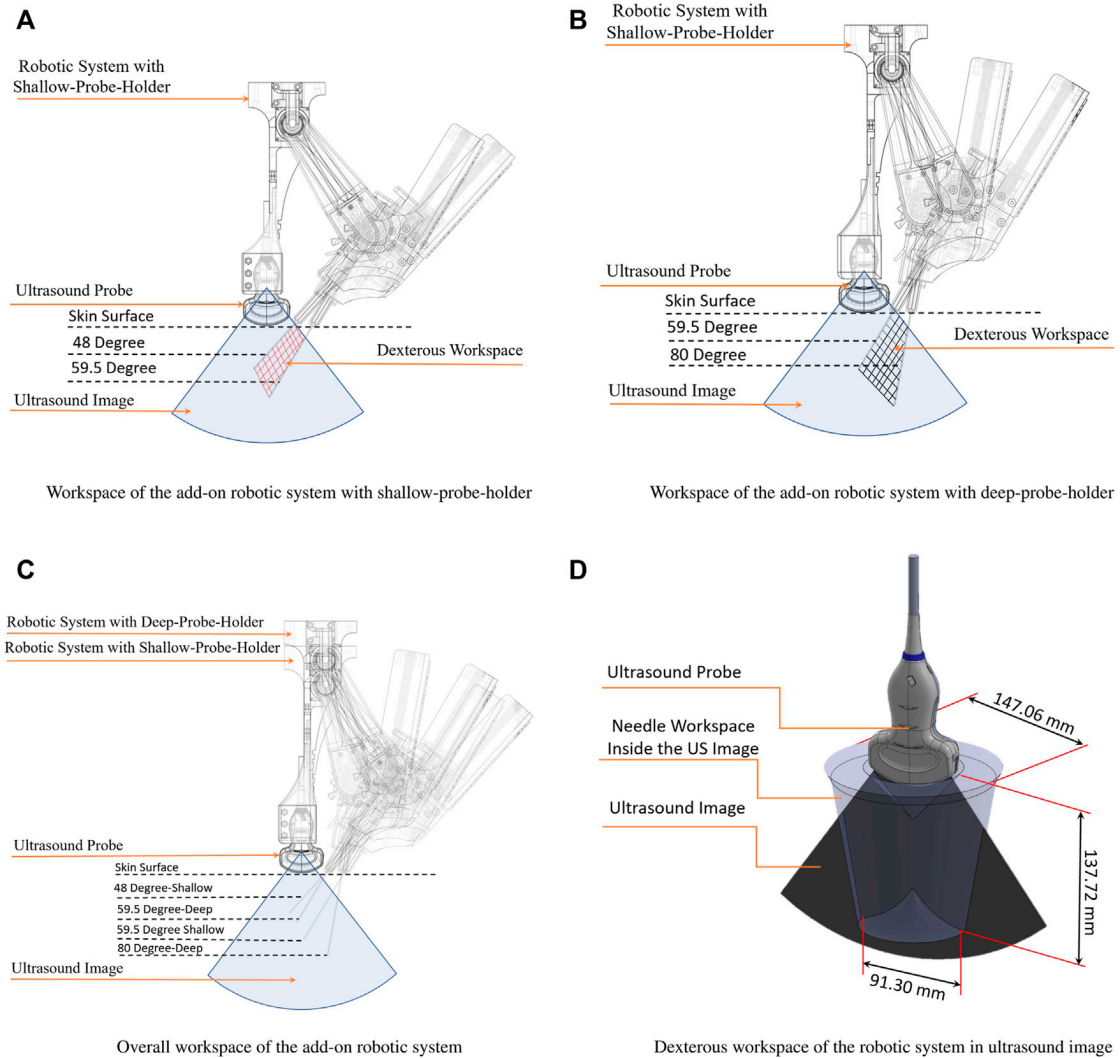
Workspace analysis of the add-on robotic system. **(A)** Workspace of the add-on robotic system with shallow-probe-holder; **(B)** Workspace of the add-on robotic system with deep-probe-holder; **(C)** Overall workspace of the add-on robotic system; **(D)** Dexterous workspace of the robotic system in ultrasound image.

In addition, we designed two probe holders with different length, shallow-probe-holder and deep-probe-holder, that can be simply mounted on the add-on robotic system. As illustrated in Figure 6A, the shallow-probe-holder covers the needle angle between 48 and 59.5° with the maximum depth 88 m. The deep-probe-holder covers the angel of 59.5–80° with the maximum depth 137 mm, as illustrated in Figure 6B. In summery the dexterous workspace for the needle insertion is between 48 and 80° with the depth of the 53–137 mm, as illustrated in Figure 6C. Considering the roll in the UR5 manipulator, the needle can cover a cone with the height of 53 mm and the inside angel of 48° and the outside angel of 80 with the height of 137 mm as illustrated in Figure 6D.

### 3.6 Electrical Schematic

Robotic systems rely on sensors and actuators as their primary components. There are several considerations to select proper sensors and actuators for the robotic systems that are physically interact with humans in medical applications. Actuators used in medical application are required to provide high accuracy, high resolution, safety, and repeatability. Torque, velocity, range of motion, disturbance rejection, and controllability are other important selection criteria. From the technical perspective, compact design, wiring, ease of control, and being compatible with sensors are other important factors to select an actuator for medical robotic system (Sajadi et al., 2022).

Meeting all these requirements is very challenging and causes a bottleneck in the design process. Smart actuators have recently been developed to address this challenge. These actuators are stand-alone modules that include a dc-motor, gearbox, encoder, embedded close-loop controller, and integrated electronic circuit for sensory data acquisition (Grosu et al., 2017). In addition to the servomotors, a double acting micro pneumatic cylinder with a 30 mm stroke is used as the actuator for the biopsy instrument’s firing trigger. Three types of the DYNAMIXEL actuators are used in the robotic system in this paper. The technical aspects of electrical actuators are presented in Table 1.

**TABLE 1:** Technical aspects of electrical actuators.

Actuator model	Resolution (pulse/rev)	Backlash (Degree)	Torque	Feed back	Position sensor	Communication protocol
DYNAMIXEL MX-106T	4,096	0.33	Stall	Position, Load Voltage, Temperature	Contactless absolute encoder [12Bit, 360 (°)]	TTL Serial Communication
			8.0N.m@11.1V			
			8.4N.m@12V			
			10.0N.m@14.8V			
DYNAMIXEL MX-64T	4,096	0.33	Stall	Position, Load Voltage, Temperature	Contactless absolute encoder [12Bit, 360 (°)]	TTL Serial Communication
			5.5N.m@11.1V			
			6N.m@12V			
			7.3N.m@14.8V			
DYNAMIXEL PM54-060-S250-R	526,374	0.1	Continuous	Position, Velocity	Contactless	RS485 Serial Communication
			10.1N.m@24V	Current, Temperature	Incremental encoder	
			—	Voltage, External Port	—	

**FIGURE 7:**
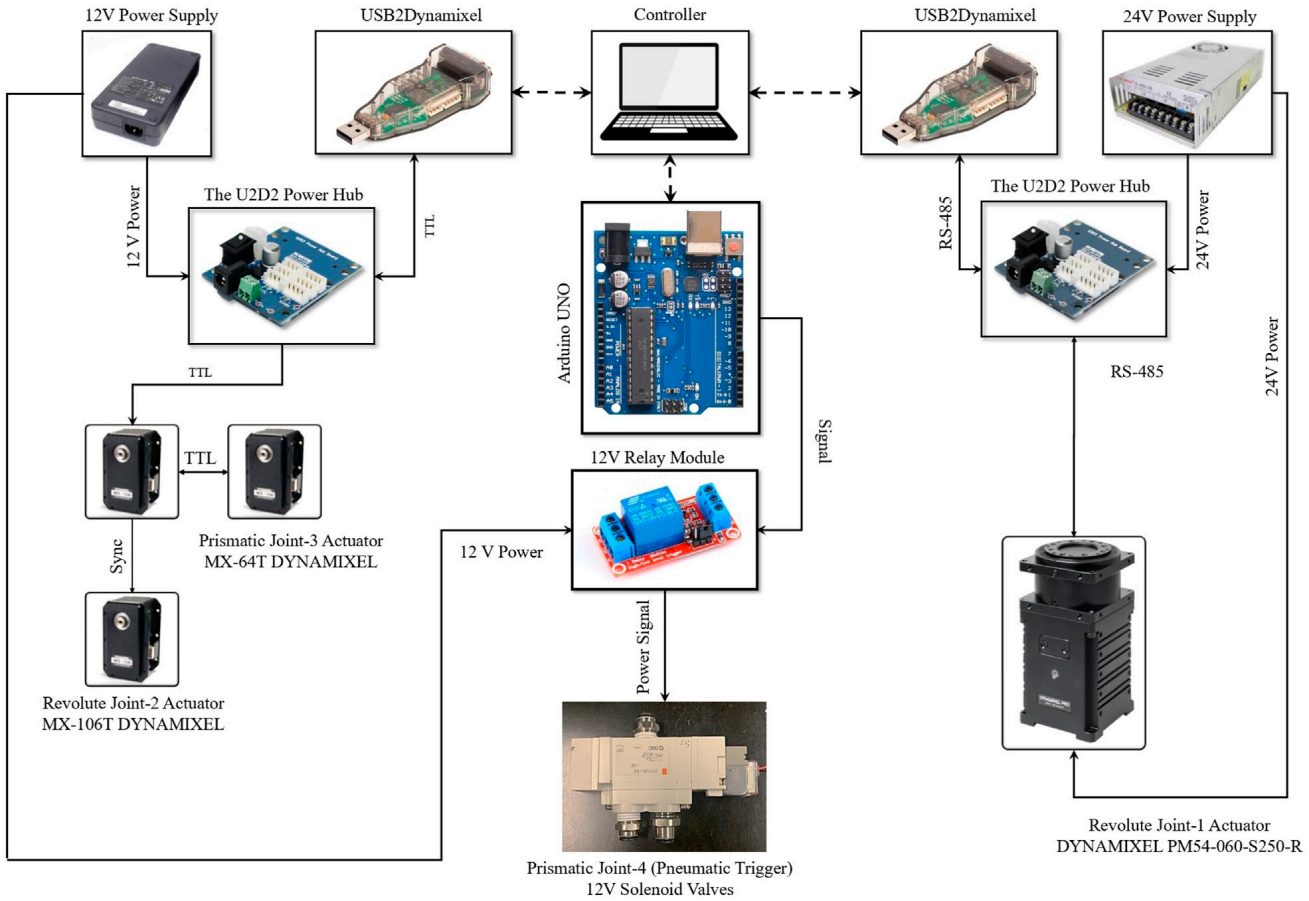
Electrical schematic.

Electrical schematic of the robotic system is illustrated in Figure 7. Smart actuators form a network with a three-wired electrical connection, which simplifies the system wiring. The RS-485 protocol is used by the DYNAMIXEL PM54-060-S250-R, whereas the TTL protocol is used by the DYNAMIXEL MX-106T and MX-64T as the communication protocols inside the motor network. Since the joint-2 has two MX-106T servomotors that operate in dual mode, a three-wired synchronization cable connects these two motors. An Arduino-UNO and a 12 V relay module control the 12 V solenoid valve that controls the pneumatic actuator.

## 4 Modeling and Control of the Add-On Robot

The add-on robot is controlled by a Reinforcement Learning (RL) agent to reach the desired position and angle, in order to prepare for inserting the needle. The RL agent is trained using Deep Deterministic Policy Gradient (DDPG) algorithm proposed by Lillicrap et al. (2010). This approach can be utilized to train RL agents with continuous action spaces and has been used in robotic applications in recent years.

The architecture of the reinforcement learning algorithm consists of an agent and an environment where the agent interacts with. The agent takes actions at each time step and receives observation *O*(*t*) and reward *r*(*t*) from the environment. The agent should be trained in a way to take actions at each state to maximize the cumulative rewards received over every episode of training.

### 4.1 The Actor and Critic Networks

**FIGURE 8:**
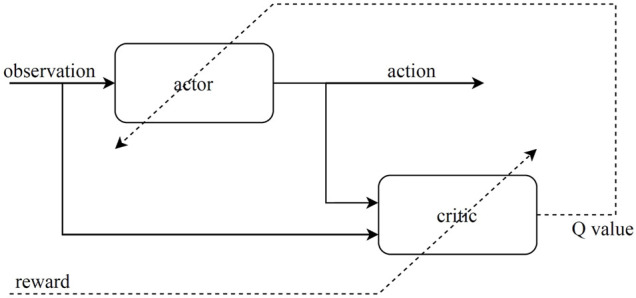
Architecture of the actor-critic reinforcement learning.

The DDGP algorithm uses an actor network which learns to choose actions by receiving *O*(*t*) from the environment, and a critic network which estimates the q values. The critic network is trained using the reward signal given by the environment at each time step, and the actor network is trained by the estimated value by the critic network as illustrated in Figure 8.

For the actor, we used a feedforward neural network consisting of two hidden layers with 400 neurons for each layer, and the *reLu* activation function. The input of the actor network is the observation signal given by the environment. The outputs of the actor are the actions. The actions taken by the controller are the angular velocity of each motor $\left(\dot{\theta_{1}},\dot{\theta_{2}}\right)$ . Thus, the output layer of the actor network consists of two neurons. Since the output of the network should be a continuous signal, we used tanh activation function for the output layer.

The critic network receives both the actions and the observations as input. Thus, the network needs two separate channels to process the input information and concatenate them later. For the observations we used two hidden layers with 30 and 70 neurons for each, and for the actions we used one hidden layer with 80 neurons and after concatenations we add two more hidden layers with 400 neurons for each. The output of the critic network is the estimated value. Therefore the output layer has a single neuron. The networks have been implemented and trained using Tensorflow library available for Python Abadi et al. (2015).

### 4.2 The Environment and Reward Function

**FIGURE 9:**
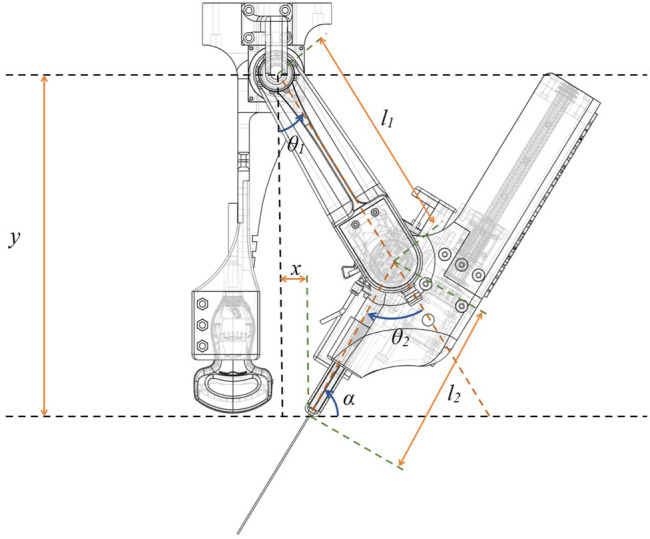
Robot model used for simulation of the environment.

For training the RL model we used a simulated environment based on the designed robot and a fixed obstacle which is assumed as the patient’s body. The robot has 3 degrees of freedom for the positioning of the needle angle (*θ*_1_, *θ*_2_, *l*_2_) actuated by two motors, which make the robot under-actuated. The control command for moving the motors is the angular velocity of the joints $\left(\dot{\theta_{1}},\dot{\theta_{2}}\right)$ . The robot model used for the simulation is a two-link planar robot with a flexible link (*l*_2_) as illustrated in Figure 9. The length of the first solid link is (*l*_1_ = 210 *mm*) and the flexible link’s minimum length is (*l*_2_min = 160 *mm*) whereas the flexible link’s maximum length is (*l*_2_max = 200 *mm*) and the stroke of the spring mechanism is (*S* = 40 *mm*).

According to the kinematic equations of the robot, the position and the angle of the needle can be calculated using the positions of the motor:

$$y=l_{1}cos\left(\theta_{1}\right)+l_{2}cos\left(\theta_{2}-\theta_{1}\right)$$

$$x=l_{1}sin\left(\theta_{1}\right)-l_{2}sin\left(\theta_{2}-\theta_{1}\right)$$

$$\alpha =\frac{\pi }{2}-\theta_{2}+\theta_{1}$$

The states of the environment are updated according to the model (robot kinematics) and the actions taken by the agent. Additionally, we used a constraint for the position of the needle in *y* direction, which represents the body of the patient. The constraint is a rigid obstacle placed in the desired *y* (*y*_*d*_) and compresses the length of the second link (*l*_2_) when the needle pushes the obstacle. The main challenge of the controller is to learn to reach the desired position and angle in the presence of the obstacle. The obstacle changes the length of the second link when it is in contact with the robot. This constraint adds an uncertainty to the environment, so the challenge of the controller is to adapt to this uncertainty.

According to the robot model, the environment has three dynamical states. Thus, the state vector is defined as:

$$S\left(t\right)=\left[\theta_{1}\left(t\right),\theta_{2}\left(t\right),l_{2}\left(t\right)\right]$$

In the experimental setup, the length of the second link is not measured directly with a sensor. But the position of the needle (*y*) is measured by a 6 DOF real-time optical tracking system. Therefore, the observation vector given to the agent includes actual position of the agent (*y*) instead of the length of the second link (*l*_2_). The angular position of the joints are measured by the encoders of the motors. The target angle of the needle (*α*_*d*_) needs to be defined by the user. Therefore, it should be a fixed variable for each episode, and also should be available to the agent. At the beginning of each episode, we initiate the value of *α*_*d*_ by a random variable in the range of the working space. Thus, the observation vector can be defined as:

$$O\left(t\right)=\left[\theta_{1}\left(t\right),\theta_{2}\left(t\right),y\left(t\right),\alpha_{d}\right]$$

For defining the reward function we need to look at the objectives of the controller. The target position and angle of the needle is fixed and determined for each episode (the desired *x* for the needle is 0 since we want the needle to be close to the probe) and can be used for calculating the target loss function:

$$loss_{1}\left(t\right)=a_{1}\sqrt{\left|\alpha \left(t\right)-\alpha_{d}\right|}+a_{2}\sqrt{\left|y\left(t\right)-y_{d}\right|}+a_{3}\sqrt{\left|x\left(t\right)\right|}$$

where *a*_*n*_ is the weight of each error. We used squared root of the absolute value of the error for each target since we want bigger punishment for the agent when the error is close to zero. In this way we improve the accuracy of the controller. We also define a second loss function in order to reduce the velocity of the motors and avoid overfitting and resonating response from the motors:

$$loss_{2}\left(t\right)={\dot{\theta_{1}}}^{2}\left(t\right)+{\dot{\theta_{1}}}^{2}\left(t\right)$$

The reward function used for training is:

$$r\left(t\right)=-\left(b_{1}loss_{1}\left(t\right)+b_{2}loos_{2}\left(t\right)\right)$$

where *b*_*n*_ is the weight of each loss function.

## 5 Experiment

### 5.1 Experimental Setup

**FIGURE 10:**
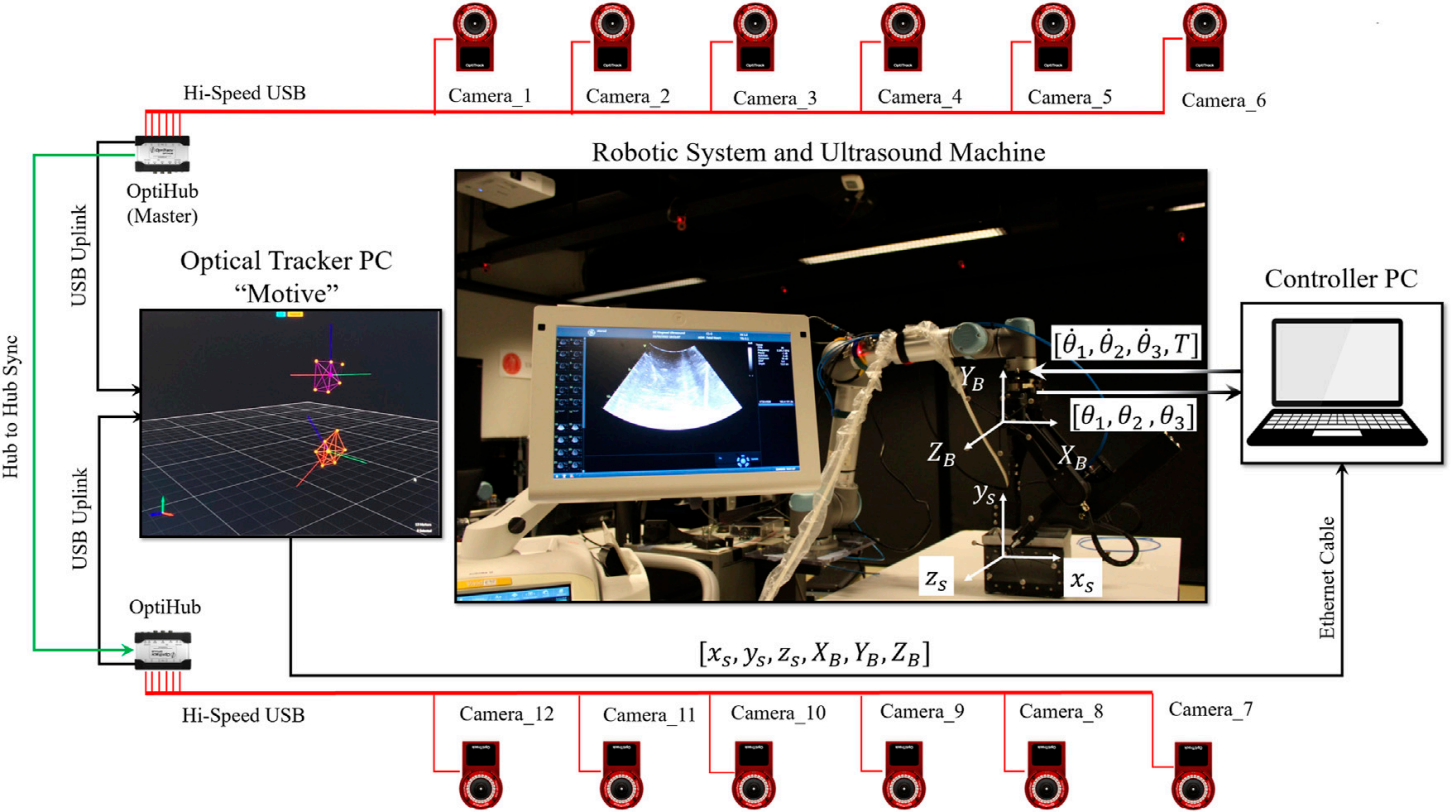
Experimental setup.

**FIGURE 11:**
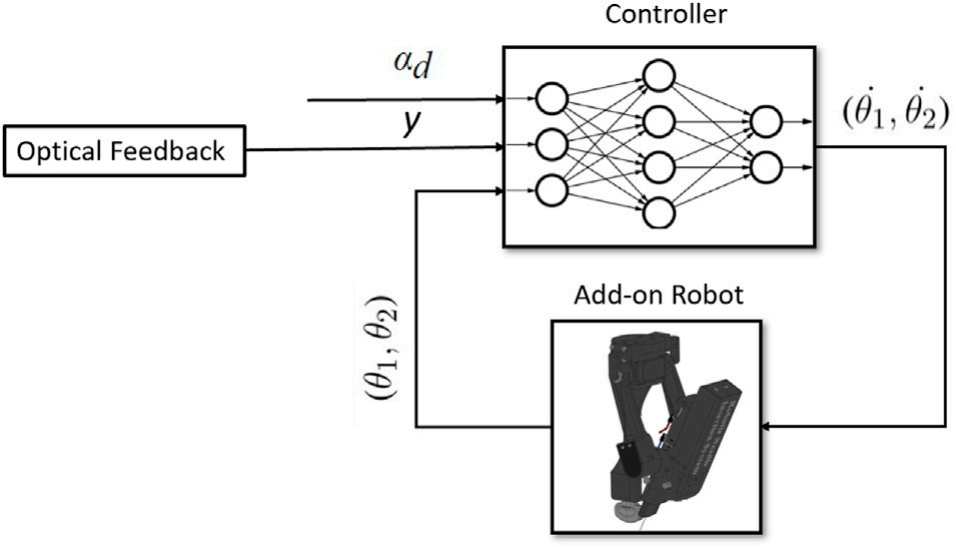
Controller implementation.

Figure 10 illustrates the experimental setup and Figure 11 illustrates the controller implementation. To measure the distance between the center of joint 1 and the end of the needle guide part, a 6 DOF real-time optical tracking system (OptiTrack) is employed. The optical tracker is equipped with 12 “Flex-13” cameras and has been calibrated with a mean error of 0.609 mm. Six of the twelve cameras are linked to the master optiHub, while the others are connected to the other optiHub through a separate high-speed USB connection for each camera. These two Hubs are linked through a USB Hub to Hub synchronization connection. These two hubs are linked to a PC, running OptiTrack’s motion capture software “Motive”, through two separate USB connection. The optical tracker latency is less than 2 ms in this setup, and the system’s feedback meets the requirements of a hard real-time system. As a result, the feedback is very well suited to the controller. Two 3D printed rigid bodies with six markers are designed to be attached to the center of joint 1 (base rigid body), and the needle guide part (needle guide rigid body) to measure the pose of these two points. The pivot point of the base rigid body is calibrated at the center of joint 1. The pivot point of the needle guide rigid body is calibrated at the end of the needle guide part. The “Motive” software is used to calibrate the pivot points of both rigid bodies. Through the NatNet client/server networking protocol, the “Motive” software streams the reconstructed 3D data (position) to the controller PC. NatNet provides a low-latency UDP connection between the optical tracker’s PC and the controller PC through an Ethernet cable. Two USB2Dynamixel interfaces link the robotic system’s smart actuators to the controller PC. The solenoids valve is controlled by the Arduino UNO, which is linked to the Controller PC through USB.

### 5.2 Training Result

**FIGURE 12:**
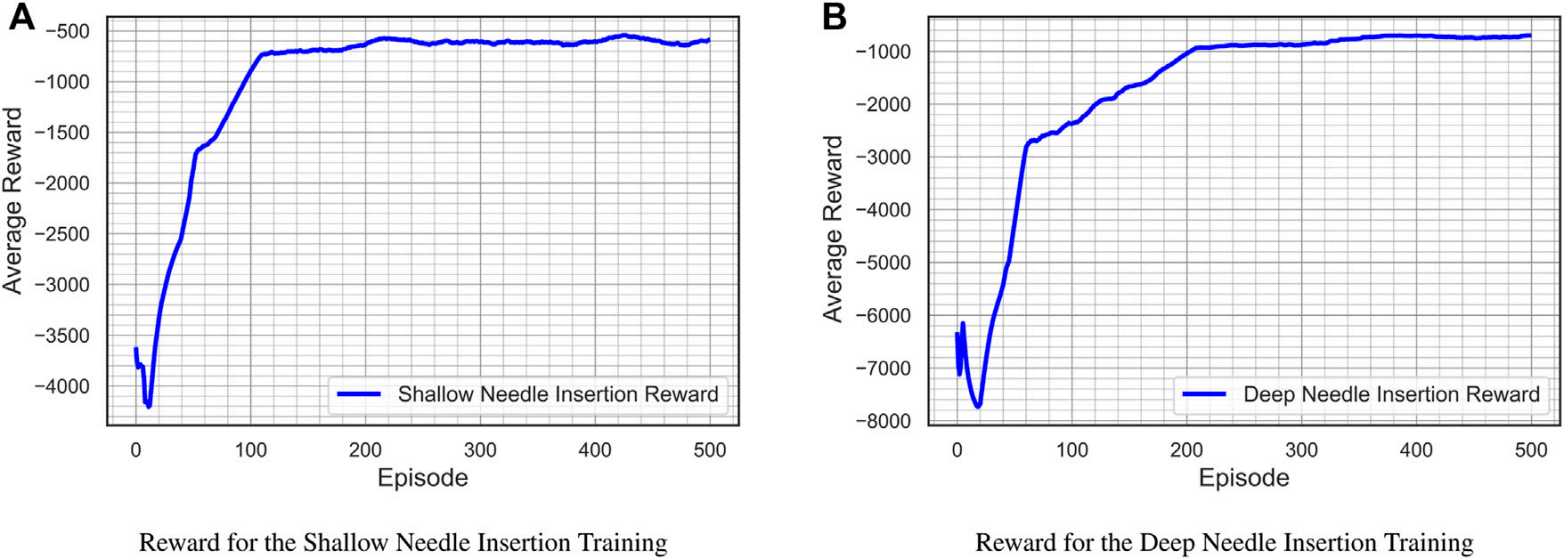
Average cumulative reward for training the control models.

The RL-agent is trained individually for deep and shallow needle insertion. The range of desired input for the shallow needle insertion is (48 < *α* < 59) whereas the range of desired input for the deep needle insertion is (59 < *α* < 70). The control model described in Section 4 was trained using TensorFlow library in python. Each network for the shallow and deep cases was trained for 500 episodes and each episode was run for 400 time-steps. Figure 12 illustrates the average cumulative reward for training of each network.

### 5.3 Experimental Result

Four comprehensive experiments were performed to evaluate the robotic system’s performance in terms of the biopsy instrument positioning, and the insertion of the needle inside the ultrasound plane. The first objective is to evaluate the robotic system’s accuracy in adjusting the needle’s angle in response to a reference input angle while maintaining the end of needle guide part at the level of the US probe for four different inputs. The second objective is to evaluate the robotic system’s performance for in-plane needle insertion in both shallow and deep insertions in a water tank. The first two experiments are dedicated to the shallow needle insertion, whereas the third and fourth experiments focus on the deep needle insertion inside of the dexterous workspace. Although there is always a gap between the simulation environment where the RL-agent is trained and the real world, the gap in this paper is very small due to the use of high resolution smart actuators in the development of the robotic system, obtaining the precise dimensions of the robotic system from the CAD model, and prototyping the system using a high precision 3D printer.

#### 5.3.1 Experiment 1-Shallow Needle Insertion

**FIGURE 13:**
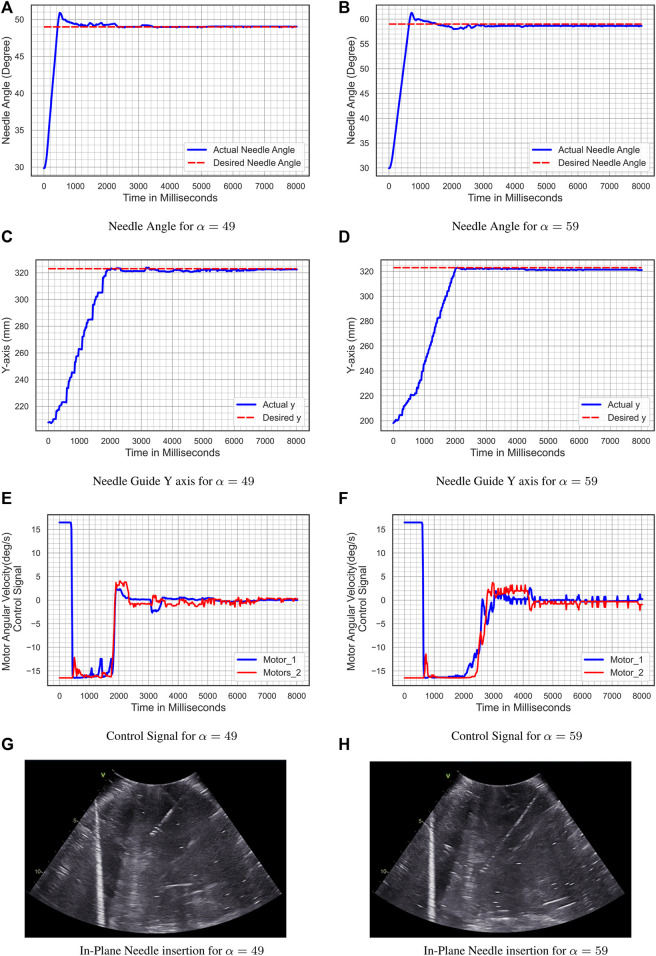
Experiment 1- shallow needle insertion. **(A)** Needle Angle for α = 49; **(B)** Needle Angle for α = 59; **(C)** Needle Guide y-axis for α = 49; **(D)** Needle Guide y-axis for α = 59; **(E)** Control Signal for α = 49; **(F)** Control Signal for α = 59; **(G)** In-Plane Needle insertion for α = 49; **(H)** In-Plane Needle insertion for α = 59.

The shallow probe holder is installed in the add-on robotic system in these two experiments. The initial conditions are *θ*_1_ (0) = 60*degree* and *θ*_2_ (0) = 120*degree*. For the first experiment, the reference input angle is *α*_1_(*t*) = 49*degree*, whereas for the second experiment, the reference input angle is*α*_2_(*t*) = 59*degree*, and the desired level is *y*_*d*_ = 323 *mm*. Figure 13 shows the result of the first experiment. Figure 13A and Figure 13B illustrate the tracking of the reference input angle, Figure 13C and Figure 13D illustrate the tracking of the desired level *y*_*d*_, Figure 13E and Figure 13F illustrate the velocity control signals, and Figure 13G and Figure 13H illustrates the in-plane needle insertion. For the reference input angle, the tracking error for *α*(*t*) = 49*°* is *e*_*α*_ = 0.148*°* and for *α*(*t*) = 59*°* is *e*_*α*_ = 0.444*°*, while for the desire level error, the tracking error for *α*(*t*) = 49*°* is *e*_*y*_ = 1.141 *mm* and for *α*(*t*) = 59*°* is *e*_*y*_ = 1.667 *mm*.

#### 5.3.2 Experiment 2-Deep Needle Insertion

**FIGURE 14:**
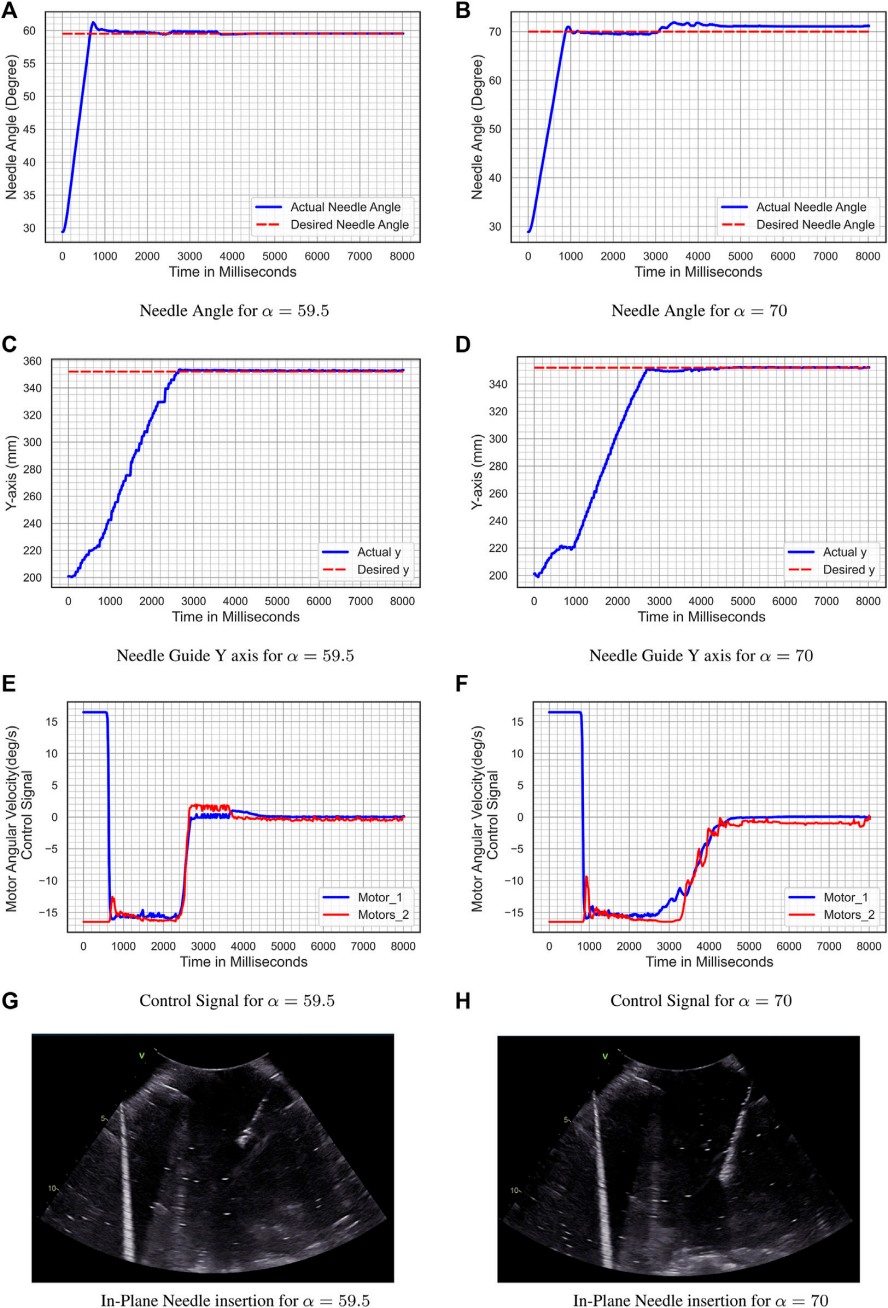
Experiment 2- deep needle insertion. **(A)** Needle Angle for α = 59.5; **(B)** Needle Angle for α = 70; **(C)** Needle Guide y-axis for α = 59.5; **(D)** Needle guide y-axis for α = 70; **(E)** Control Signal for α = 59.5; **(F)** Control Signal for α = 70; **(G)** In-Plane Needle insertion for α = 59.5; **(H)** In-Plane Needle insertion for α = 70.

The deep probe holder is installed in the add-on robotic system in these two experiments. The initial conditions are *θ*_1_ (0) = 60*°* and *θ*_2_ (0) = 120*°*. For the first experiment, the reference input angle is *α*_1_(*t*) = 59.5*°*, whereas for the second experiment, the reference input angle is*α*_2_(*t*) = 70*°*, and the desired level is *y*_*d*_ = 352 *mm*. Figure 14 shows the result of the second experiment. Figure 14A and Figure 14B illustrate the tracking of the reference input angle, Figure 14C and Figure 14D illustrate the tracking of the desired level *y*_*d*_, Figure 14E and Figure 14F illustrate the velocity control signals, and Figure 14G and Figure 14H illustrates the in-plane needle insertion. For the reference input angle, the tracking error for *α*(*t*) = 59.5*°* is *e*_*α*_ = 0.181*°* and for *α*(*t*) = 70*°* is *e*_*α*_ = 1.011*°*, while for the desire level error for *α*(*t*) = 59.5*°* is *e*_*y*_ = 0.68 *mm* and for *α*(*t*) = 70*°* is *e*_*y*_ = 0.367 *mm*.

## 6 Discussion

While several imaging modalities are employed for image-guided percutaneous needle biopsy, ultrasound is the most often used image modality. ultrasound is more extensively utilized due to its real-time imaging, mobility, low cost, ease of use, low risk of side effects, and lack of ionizing radiation exposure. A successful percutaneous needle biopsy requires precise needle insertion into the lesion. A discrepancy between the biopsy’s specimen and the target lesion due to incorrect biopsy needle insertion can lead to misinterpretation and errors in the diagnosis resulting in false negatives and adverse consequences. Furthermore, the size and quality of the specimen are critical for the diagnostic informative value of a biopsy procedure to reduce the risk of misinterpretation and enhance the inter-observer variability.

While the end cut full-core needle instrument has been recently developed to improve the size and quality of biopsy’s specimen, image-guided percutaneous needle biopsies are currently performed manual. The procedure’s quality is determined by the radiologist’s expertise, precision, and dexterity. Moving toward an ultrasound-guided autonomous robotic biopsy based on the end cut full-core needle instrument to enhance the precision of the needle insertion is an ideal solution to this shortcoming. Such a robotic system can lead to a valuable biopsy to gain as much tissue as possible with the smallest possible trauma. The initial step toward achieving this objective is to develop a fully automated robotic system based on a commercial full-core biopsy instrument. The system should have sufficient degrees of freedom to meet clinical requirements while also covering a reasonably large dexterous workspace inside the ultrasound image. Such a system can also be utilized as a slave robot in a long-distance teleoperation configuration.

The developed robotic system in this paper consists of a novel fully automate robot that has been customized for a BioPince ultra full core biopsy instrument and attached to a UR5-based teleoperation system with 6 DOF. The dexterous workspace of the add-on robotic system is detailed in Section 3.4, and it is demonstrated that the system has a suitable workspace within the ultrasound image. The UR5 manipulator has been controlled by a radiologist utilizing a master-slave configuration and a human-in-the-loop control strategy to manipulate the ultrasound probe and the add-on robotic system. As stated in the authors’ previous research in (Mathiassen et al., 2016), the teleoperation system equipped with a 6 DOF force/torque sensor for the force control mode, in which the force controller maintains a constant pressure between the ultrasound probe and the patient’s body. As a result, the teleoperation system is pretty stable to the ultrasound shadowing.

Since one of the objectives of this research was preparing the system for an envisioned autonomous robotic biopsy based on the ultrasound image, the add-on robotic system is controlled by a reinforcement learning-based controller. The reinforcement learning agent has been trained using the Deep Deterministic Policy Gradient (DDPG) algorithm that is suitable for continuous action spaces. To prepare the biopsy instrument for needle insertion, the angular velocity command controls the desired position and angle of the biopsy instrument. The radiologist uses the robotic system to insert the needle, ensuring that a human is still in charge throughout the invasive step. The overall mean error of all four experiments in the tracking of the needle angle is 0.446° and the resolution of the needle insertion is 0.002 mm.

Since developing a light-weight robotic system was one of the objectives of this research, the add-on robot should be prototyped with the weight of less than 5 kg (4.28 kg) to be able to handle by the UR5 manipulator. As a result of this consideration, the add-on robotic system lacks one degree of freedom, which is covered by adjustable link-2 with the novel spring mechanism and design two probe holders for shallow and deep insertion. Four experiments in Section 5 verified the system’s ability to insert the needle within the US image (probe-guided biopsy) in a rather large workspace (free-hand biopsy).

While the whole robotic system was 3D printed, several major elements such as the biopsy instrument, ultrasound probe, actuators, ball-screw, and linear slider were purchased commercially with predetermined weights and dimensions. Thus, the overall weight and size of the add-on robotic system can be reduced by customizing the size of these elements, particularly the full core biopsy instrument. In this way the mechanical design can be modified with one additional degree of freedom to increase the flexibility of the robotic system. However, such a system can no longer use a commercial full core instrument and requires a specially developed biopsy tool which may have a lower needle design quality.

## 7 Conclusion and Future Work

A novel fully automated robotic-assisted system for the positioning and insertion of a commercial end cut full core biopsy instrument has been developed in this paper. The robotic system has been composed of a 4 DOF add-on robot for a commercial full core biopsy instrument and attached to a UR5-based teleoperation system with 6 DOF. The proposed robotic system can be used as a slave robot in a teleoperation configuration or as an autonomous or semi-autonomous robot in the future. The dexterous workspace analysis of the add-on robotic system demonstrated that the system has a suitable workspace within the US image.

While the UR5 manipulator was controlled using a teleoperated system designed by the authors earlier in (Mathiassen et al., 2016), the add-on robot has been controlled by an RL based controller using the Deep Deterministic Policy Gradient (DDPG) algorithm. The RL controller has been used to position the biopsy instrument in order to get it ready for needle insertion. High resolution needle insertion has been done by robotic system under the supervision of the radiologist, ensuring that a human is still in charge throughout the invasive step. The robotic system’s performance in terms of biopsy instrument positioning and needle insertion inside the ultrasonic plane has been evaluated in four comprehensive experiments. The experimental results showed the ability of the robotic system for in-plane needle insertion. The overall mean error of all four experiments in the tracking of the needle angle was 0.44618°, and the resolution of the needle insertion was 0.002 mm.

Future work on this system will improve the mechanical design by integrating a length measurement sensor in the spring mechanism to measure the length of the link 2. The RL controller will be upgraded to train for autonomous needle insertion based on US image. Long-distance teleoperation of the system will be achieved by the system’s integration with a 5G network and augmented reality, which will allow for holographic visualization of the procedure.

## Data Availability

The original contributions presented in the study are included in the article/Supplementary Material, further inquiries can be directed to the corresponding author.
